# Vortioxetine Subchronically Activates Serotonergic Transmission via Desensitization of Serotonin 5-HT_1A_ Receptor with 5-HT_3_ Receptor Inhibition in Rats

**DOI:** 10.3390/ijms20246235

**Published:** 2019-12-10

**Authors:** Motohiro Okada, Ruri Okubo, Kouji Fukuyama

**Affiliations:** Department of Neuropsychiatry, Division of Neuroscience, Graduate School of Medicine, Mie University; Tsu 514-8507, Japan; ddduck0602@gmail.com (R.O.); k-fukuyama@clin.medic.mie-u.ac.jp (K.F.)

**Keywords:** vortioxetine, escitalopram, MK801, serotonin, GABA, microdialysis, depression, rat

## Abstract

Vortioxetine is a novel, multimodal antidepressant with unique targets, including the inhibition of the serotonin transporter (SET), of serotonin 5-HT_3_ (5-HT3R), and of 5-HT_7_ (5-HT7R) receptors and partial agonism to serotonin 5-HT_1A_ (5-HT1AR) receptors in humans. Vortioxetine has a lower affinity to 5-HT1AR and 5-HT7R in rats compared with humans, but several behavior studies have demonstrated its powerful antidepressant-like actions. In spite of these efforts, detailed effects of the subchronic administration of vortioxetine on serotonergic transmission remain to be clarified. This study examined the mechanisms underlying the clinical effects of vortioxetine by measuring the releases of 5-HT and GABA in the medial prefrontal cortex (mPFC) of freely moving rats compared with the selective SET inhibitor, escitalopram. Inhibition of 5-HT3R in the mPFC enhanced regional 5-HT release via GABAergic disinhibition. Activation of somatodendritic 5-HT1AR in the dorsal raphe nucleus (DRN) and presynaptic 5-HT1AR in the mPFC inhibited 5-HT release in the mPFC. Escitalopram subchronically activated mesocortical serotonergic transmission via desensitization of 5-HT1AR in the mPFC and DRN and of 5-HT3R in the mPFC; however, vortioxetine also subchronically activated mesocortical serotonergic transmission via desensitization of 5-HT1AR in the mPFC and DRN but not of 5-HT3R in the mPFC. These demonstrations, the desensitization of 5-HT1AR with the inhibition of 5-HT3R (without 5-HT3R desensitization), at least partially, contribute to the multimodal antidepressant action of vortioxetine in rats.

## 1. Introduction

Vortioxetine, which is classified as “N06AX26” in the “N06AX other, antidepressants class” by the anatomical therapeutic classification system of the World Health Organization, was approved by the Food and Drug Administration in 2012 [1], the European Medicines Agency in 2013 [2], and the Pharmaceuticals and Medical Devices Agency (Japan) in 2019 for the treatment of major depressive disorders [1]. The mechanisms of antidepressive action of vortioxetine are considered to be its unique serotonergic binding profiles, i.e., inhibition of the serotonin (5-HT) transporter (SET) with modulation of several types of 5-HT receptor subtypes [3]. Based on the binding profiles of vortioxetine, this new type of antidepressant is classified as a serotonin partial agonist reuptake inhibitor (SPARI) by the European College of Neuropsychopharmacology [4].

Various standard behavioral studies, including a forced swim test, a social interaction test, a conditioned fear-induced vocalization test, a novelty-suppressed feeding test, and an open field test, have demonstrated its effectiveness against depressive and anxiety disorders [3,5]. Furthermore, the potentials of the cognitive enhancing action of vortioxetine have also been demonstrated [6,7]. Notably, vortioxetine reversed the glutamate/*N*-methyl-d-aspartate (NMDA)/glutamate receptor (NMDA-R) impairment-induced deficits in the extra-dimensional part of the set-shifting test [8].

The major mechanisms of the multimodal action of vortioxetine are considered to be the inhibition of the serotonin 5-HT_3_ (5-HT3R) and 5-HT_7_ (5-HT7R) receptors and SET, and the partial agonism to the serotonin 5-HT_1A_ receptor (5-HT1AR) [9], since several 5-HT receptor subtypes—5-HT1AR, 5-HT3R, 5-HT7R, and SET—relate to cognitive functions [10]. The binding profiles of vortioxetine using recombinant cell lines expressed in human and rat targets were not identical [11,12]. These studies demonstrated that vortioxetine was a high-affinity inhibitor of human 5-HT3R (Ki = 3.7 nM), 5-HT7R (Ki = 19 nM), and SET (1.6 nM) and an agonist of 5-HT1AR (Ki = 15 nM), whereas vortioxetine also had a high affinity to rat 5-HT3R (Ki = 1.1 nM) and SET (Ki = 8.6 nM) but a lower affinity to 5-HT1AR (Ki = 230 nM) or 5-HT7R (Ki = 200 nM) [11,12]. The lower affinity of vortioxetine to 5-HT1AR and 5-HT7R in rats compared to humans may mean that the net pharmacodynamic effect of vortioxetine mediated through these receptors is underestimated in rat behavior studies. In other words, the effects of vortioxetine on rodent behavior are predominantly affected by the inhibition of 5-HT3R and SET rather than 5-HT1AR and 5-HT7R [11,13].

It has been well established that the downregulation/desensitization of 5-HT1AR plays important roles in the antidepressive mechanisms of several classes of antidepressants, including the selective SET inhibitor, the serotonin/norepinephrine transporter inhibitor, and mirtazapine [14,15,16]. 5-HT1AR, which is expressed in serotonergic neurons in the dorsal raphe nucleus (DRN), suppresses serotonergic neuronal activity via activation of inwardly rectifying potassium channels [16]. In contrast to 5-HT1AR, 5-HT3R is a nonselective, ligand-gated cation channel that generates rapid neuronal depolarization of neurons [17]. 5-HT3R expresses in the cortex but not in the mediodorsal thalamic nucleus (MDTN) or DRN [18]. Interestingly, impaired 5-HT3R enhances the effects of a low dose of citalopram (selective SET inhibitor) [19]. Additionally, behavior studies using ondansetron (selective 5-HT3R antagonist) have indicated its antidepressive-like effects [20] and have suggested the interaction between 5-HT3R and NMDA-R [20].

A noncompetitive NMDA-R antagonist, esketamine, was approved by the FDA in early 2019 as a rapid-acting antidepressant for the treatment of major depressive disorders [21]. Indeed, monoaminergic antidepressants have delayed the onset of antidepressive actions and take up to several weeks to exert their salutary effects; however, single doses of ketamine have superior response rates, within hours to days, of administration [22]. Contrary to the effectiveness of esketamine/ketamine, NMDA-R dysfunction is implicated in the pathophysiology of schizophrenia, as evidenced by the induction of schizophrenia-like cognitive impairment, positive and negative symptoms in healthy volunteers and experimental animal models, and the exacerbation of psychosis in schizophrenia patients by noncompetitive NMDA-R antagonists such as phencyclidine and ketamine [23,24]. We have demonstrated that the hyperactivation of thalamocortical glutamatergic transmission induced by the NMDA-R antagonist plays an important role in the cognitive impairment in schizophrenia [25,26,27,28].

In light of these above findings, the present study was designed to elucidate the actions of vortioxetine on serotonergic transmission within the mesocortical serotonergic pathway, from the DRN to the medial prefrontal cortex (mPFC). For this purpose, the following experiments were conducted in freely moving rats using multiprobe microdialysis with ultra-high-performance liquid chromatography (UHPLC): (1) determination of the acute effects of systemic administration of vortioxetine on transmission of 5-HT and GABA compared with an SET inhibitor, escitalopram; (2) determination of the effects of subchronic systemic administration of vortioxetine on activities of 5-HT1AR and 5-HT3R compared with escitalopram; and (3) determination of the effects of subchronic administration of vortioxetine on mesocortical serotonergic transmission induced by MK801 (selective NMDA-R antagonist).

## 2. Results

Rats were randomly assigned to the various treatment groups specified in each study design. The present study used four study designs distinguished by the drug administration route.

### 2.1. Effects of the Interaction between the Local Administration of 5-HT3R Agents (Ondansetron and SR57227) into the mPFC and the Acute Systemic Administrations of Effective Doses of Antidepressants (Escitalopram and Vortioxetine) on the Extracellular Levels of 5-HT and GABA in the mPFC (Study-1)

**Figure 1:**
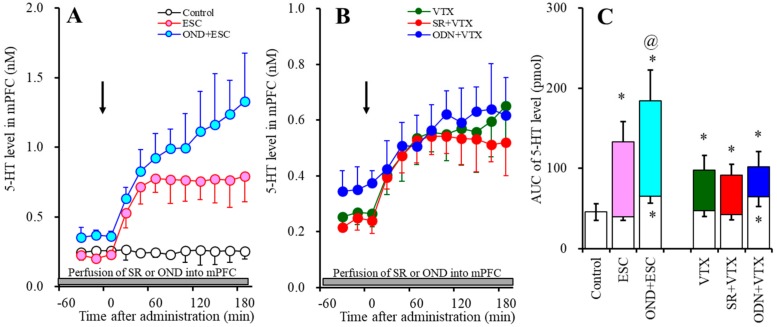
Effects of the interaction between local administration of 5-HT3R agents (ondansetron and SR57227) into the medial prefrontal cortex (mPFC) and acute systemic administrations of effective doses of antidepressant (escitalopram and vortioxetine) on the extracellular 5-HT level in the mPFC. (**A**,**B**) indicate the effects of intraperitoneal administrations of effective doses of escitalopram (ESC: 5 mg/kg) and vortioxetine (VTX: 2.5 mg/kg) on the extracellular 5-HT level in the mPFC, respectively. Effects of perfusion with 10 µM SR57227 (SR: 5-HT3R agonist) and 10 µM ondansetron (OND: 5-HT3R antagonist) on escitalopram- and vortioxetine-induced elevation of the extracellular 5-HT level in the mPFC are also indicated in (**A**,**B**). Ordinates: mean ± SD (*n* = 6) of the extracellular 5HT level (nM); abscissa: time after administration of escitalopram or vortioxetine (min). Arrows indicate intraperitoneal administration of escitalopram or vortioxetine. Gray bars indicate perfusion with SR57227 or ondansetron into the mPFC. Microdialysis was conducted to measure the releases of 5-HT and GABA. (**C**) indicates the area under the curve (AUC) value of the extracellular 5-HT level (pmol) after intraperitoneal administration of escitalopram or vortioxetine from 20 to 180 min, based on (**A**,**B**). Opened columns represents the AUC values before administration of escitalopram or vortioxetine. * *p* < 0.05, relative to control (in (**A**)), and ^@^ *p* < 0.05, relative to escitalopram (ESC) by MANOVA with Tukey’s post hoc test.

**Figure 2:**
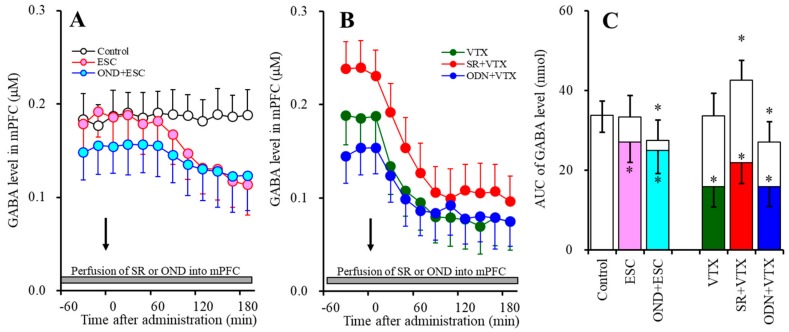
Effects of the interaction between the local administration of 5-HT3R agents (ondansetron and SR57227) into the mPFC and the acute systemic administrations of effective doses of antidepressants (escitalopram and vortioxetine) on the extracellular GABA level in the mPFC. (**A**,**B**) indicate the effects of the intraperitoneal administrations of effective doses of escitalopram (ESC: 5 mg/kg) and vortioxetine (VTX: 2.5 mg/kg) on the extracellular GABA level in the mPFC. Effects of perfusion with 10 µM SR57227 (SR: 5-HT3R agonist) and 10 µM ondansetron (OND: 5-HT3R antagonist) on escitalopram- and vortioxetine-induced reduction of GABA in the mPFC are also indicated in (**A**,**B**). Ordinates: mean ± SD (*n* = 6) of the extracellular GABA level (µM); abscissa: time after administration of escitalopram or vortioxetine (min). Arrows indicate the intraperitoneal administration of escitalopram or vortioxetine. Gray bars indicate perfusion with SR57227 or ondansetron into the mPFC. Microdialysis was conducted to measure the releases of 5-HT and GABA. (**C**) indicates the AUC value of the extracellular GABA level (nmol) after intraperitoneal administration of escitalopram or vortioxetine from 20 to 180 min, based on (**A**,**B**). Opened columns represent the AUC values before the administration of escitalopram or vortioxetine. * *p* < 0.05, relative to control (in (**A**)).

Study-1 was designed to determine the effects of acute systemic administrations of an effective dose of vortioxetine (2.5 mg/kg) [5] on extracellular levels of 5-HT and GABA in the mPFC, compared with that of the selective SET inhibitor, an effective dose of escitalopram (5 mg/kg) [29]. A perfusion medium in the mPFC was commenced with modified Ringer’s solution (MRS). After confirming the stabilization of extracellular levels of 5-HT and GABA in the perfusate, rats were treated with vortioxetine or escitalopram intraperitoneally (Figure 1 and Figure 2). Additionally, to clarify the effects of the 5-HT3R antagonism of vortioxetine on the extracellular levels of 5-HT and GABA, a perfusion medium in the mPFC was commenced with MRS containing SR57227 (10 μM: 5-HT3R agonist) [30] or ondansetron (10 μM: 5-HT3R antagonist) [31]. After confirming the stabilization of extracellular levels of 5-HT and GABA in the perfusate, rats were treated with vortioxetine or escitalopram intraperitoneally (Figure 1 and Figure 2).

#### 2.1.1. Effects of the Interaction between the Local Administration of 5-HT3R Agents (Ondansetron and SR57227) into the mPFC and the Acute Systemic Administrations of Effective Doses of Antidepressants (Escitalopram and Vortioxetine) on the Extracellular 5-HT Level in the mPFC

Acute systemic administration of an effective dose of escitalopram (5 mg/kg) increased the extracellular 5-HT level in the mPFC (Figure 1A,C). Perfusion with 10 µM ondansetron (5-HT3R antagonist) into the mPFC increased the regional extracellular 5-HT level (Figure 1A,C), whereas perfusion with 10 µM SR57227 (5-HT3R agonist) had no effect (Figure 1B,C). Perfusion with 10 µM ondansetron into the mPFC enhanced the increase in the level of extracellular 5-HT in the mPFC induced by systemic escitalopram administration (F_ODN_ (2, 15) = 38.5 (*p* < 0.05), F_Time_ (3.0, 44.6) = 55.6 (*p* < 0.05), F_ODN*Time_ (5.9, 44.6) = 19.1 (*p* < 0.05)) (Figure 1A,C).

Acute systemic administration of an effective dose of vortioxetine (2.5 mg/kg) increased the extracellular 5-HT level in the mPFC (Figure 1B,C). Contrary to escitalopram, perfusion with 10 µM ondansetron into the mPFC did not affect the vortioxetine-induced extracellular 5-HT elevation in the mPFC (F_ODN_ (2, 15) = 20.9 (*p* < 0.05), F_Time_ (4.1, 61.1) = 48.8 (*p* < 0.05), F_ODN*Time_ (8.2, 61.1) = 14.6 (*p* < 0.05)) (Figure 1B,C). Perfusion with 10 µM SR57227 into the mPFC also did not affect the vortioxetine-induced extracellular 5-HT elevation in the mPFC (F_SR_ (2, 15) = 20.8 (*p* < 0.05), F_Time_ (4.7, 70.7) = 44.1 (*p* < 0.05), F_SR*Time_ (9.4, 60.7) = 12.8 (*p* < 0.05)) (Figure 1B,C).

#### 2.1.2. Effects of the Interaction between the Local Administration of 5-HT3R Agents (Ondansetron and SR57227) into the mPFC and the Acute Systemic Administrations of Effective Doses of Antidepressants (Escitalopram and Vortioxetine) on the Extracellular GABA Level in the mPFC

Contrary to 5-HT, acute systemic administrations of effective doses of both escitalopram (5 mg/kg) and vortioxetine (2.5 mg/kg) decreased the extracellular GABA level in the mPFC (Figure 2). Perfusion with 10 µM ondansetron into the mPFC decreased the regional extracellular GABA level, whereas perfusion with 10 µM SR57227 increased the regional GABA level (Figure 2).

Perfusion with 10 µM ondansetron into the mPFC did not affect the reduction of the extracellular GABA level in the mPFC induced by systemic escitalopram administration (F_ODN_ (2, 15) = 4.2 (*p* < 0.05), F_Time_ (5.5, 82.5) = 52.2 (*p* < 0.05), F_ODN*Time_ (11.0, 82.5) = 16.7 (*p* < 0.05)) (Figure 2A,C). Similar to escitalopram, perfusion with 10 µM ondansetron into the mPFC did not affect the reduction of the extracellular GABA level in the mPFC induced by systemic vortioxetine administration (F_ODN_ (2, 15) = 23.0 (*p* < 0.05), F_Time_ (9, 135) = 105.8 (*p* < 0.05), F_ODN*Time_ (18, 135) = 48.4 (*p* < 0.05)) (Figure 2B,C). Perfusion with 10 µM SR57227 into the mPFC also did not affect the reduction of the extracellular GABA level in the mPFC induced by systemic vortioxetine administration (F_SR_ (2, 15) = 16.0 (*p* < 0.05), F_Time_ (9, 135) = 173.3 (*p* < 0.05), F_SR*Time_ (18, 135) = 48.4 (*p* < 0.05)) (Figure 2B,C).

### 2.2. Effects of Subchronic Systemic Administrations of Escitalopram and Vortioxetine on the Functions of 5-HT1AR and 5-HT3R in the mPFC and on Extracellular Levels of 5-HT and GABA in the mPFC (Study-2)

**Figure 3:**
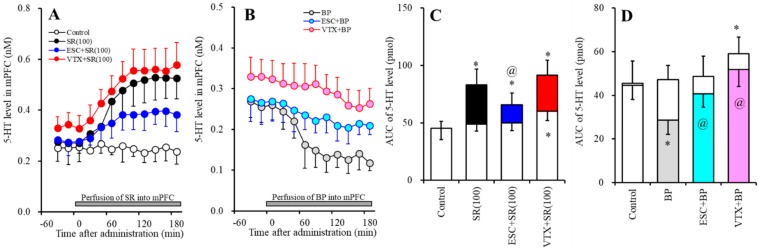
Effects of subchronic systemic administrations of escitalopram and vortioxetine on 5-HT release in the mPFC mediated by the functions of 5-HT3R and 5-HT1AR in the mPFC. (**A**,**B**) indicate the effects of perfusion with 100 µM SR57227 (SR: 5-HT3R agonist) and 50 µM BP554 (BP: 5-HT1AR agonist) on the regional extracellular 5-HT level, after the subchronic systemic administrations of escitalopram and vortioxetine, respectively. Ordinates: mean ± SD (*n* = 6) of the extracellular 5HT level (nM); abscissa: time after administration of SR57227 or BP554 (min). Gray bars indicate perfusion with SR57227 or BP554 into the mPFC. Microdialysis was conducted to measure the releases of 5-HT and GABA. (**C**,**D**) indicate the AUC value of the extracellular 5-HT level (pmol) after perfusion with SR57227 or BP554 from 20 to 180 min, based on (**A**,**B**), respectively. Opened columns represent the AUC values before perfusion with SR57227 or BP554. * *p* < 0.05, relative to control (in (**A**)), and ^@^ *p* < 0.05, relative to SR57227 (in (**A**)) or BP554 (**B**) by MANOVA with Tukey’s post hoc test.

**Figure 4:**
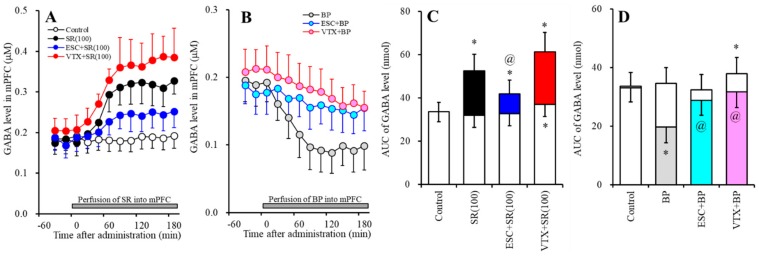
Effects of subchronic systemic administrations of escitalopram and vortioxetine on GABA release in the mPFC mediated by functions of 5-HT3R and 5-HT1AR in the mPFC. (**A**,**B**) indicate the effects of perfusion with 100 µM SR57227 (SR: 5-HT3R agonist) and 50 µM BP554 (BP: 5-HT1AR agonist) on the regional extracellular GABA level, after the subchronic systemic administrations of escitalopram and vortioxetine, respectively. Ordinates: mean ± SD (*n* = 6) of the extracellular GABA level (µM); abscissa: time after administration of SR57227 or BP554 (min). Gray bars indicate perfusion with SR57227 or BP554 into the mPFC. Microdialysis was conducted to measure the releases of 5-HT and GABA. (**C**,**D**) indicate the AUC value of the extracellular GABA level (nmol) after perfusion with SR57227 or BP554 from 20 to 180 min, based on (**A**) and (**B**), respectively. Opened columns represent the AUC values before perfusion with SR57227 or BP554. * *p* < 0.05, relative to control (in (**A**)), and ^@^ *p* < 0.05, relative to SR57227 (in (**A**)) or BP554 (in (**B**)) by MANOVA with Tukey’s post hoc test.

Study-2 was designed to determine the effects of subchronic subcutaneous administrations of effective doses of escitalopram (5 mg/kg/d) and vortioxetine (2.5 mg/kg/d) for 7 d using an osmotic pump (2ML_1, Alzet, Cupertino, CA, USA) on serotonergic and GABAergic transmissions associated with 5-HT1AR and 5-HT3R in the mPFC. The rat elimination half-lives of subcutaneous administrations of vortioxetine and escitalopram are approximately 8 and 2 h, respectively [29,32]. Based on these pharmacokinetic data, after a 24 h stop of subchronic administrations of escitalopram and vortioxetine, a perfusion medium in the mPFC was commenced with MRS. After confirming the stabilization of extracellular levels of 5-HT and GABA in the perfusate, the perfusate in the mPFC was switched to MRS containing SR57227 (100 μM: 5-HT3R agonist) [30] or BP554 (50 μM: 5-HT1AR agonist) [14,15,33] (Figure 3 and Figure 4).

#### 2.2.1. Effects of Subchronic Systemic Administrations of Escitalopram and Vortioxetine on the Functions of 5-HT1AR and 5-HT3R in the mPFC and on the Extracellular 5-HT Level in the mPFC

The subchronic systemic administration of escitalopram (5 mg/kg/d for 7 d) did not affect the extracellular 5-HT level in the mPFC (Figure 3A,C). Perfusion with 100 µM SR57227 into the mPFC increased the regional extracellular 5-HT level, whereas the stimulatory effects of perfusion with 100 µM SR57227 into the mPFC were reduced by subchronic escitalopram administration (F_SR_ (2, 15) = 17.8 (*p* < 0.05), F_Time_ (5.8, 87.3) = 74.9 (*p* < 0.05), F_SR*Time_ (11.6, 87.3) = 31.4 (*p* < 0.05)) (Figure 3A,C). Perfusion with 50 µM BP554 (5-HT1AR agonist) into the mPFC decreased the regional extracellular 5-HT level, whereas the inhibitory effect of BP554 was reduced by the subchronic administration of escitalopram (F_BP_ (2, 15) = 7.5 (*p* < 0.05), F_Time_ (9, 135) = 39.6 (*p* < 0.05), F_BP*Time_ (18, 135) = 12.4 (*p* < 0.05)) (Figure 3B,D).

The subchronic systemic administration of vortioxetine (2.5 mg/kg) weakly increased the extracellular 5-HT level in the mPFC (Figure 3A,C). The stimulatory effect of perfusion with 100 µM SR57227 into the mPFC was not affected by subchronic vortioxetine administration (F_SR_ (2, 15) = 25.9 (*p* < 0.05), F_Time_ (9, 135) = 109.8 (*p* < 0.05), F_SR*Time_ (18, 135) = 31.7 (*p* < 0.05)) (Figure 3A,C); however, the inhibitory effect of BP554 was reduced by the subchronic administration of vortioxetine (F_BP_ (2, 15) = 14.9 (*p* < 0.05), F_Time_ (5.3, 80.1) = 41.1 (*p* < 0.05), F_BP*Time_ (10.7, 80.1) = 14.8 (*p* < 0.05)) (Figure 3B,D).

#### 2.2.2. Effects of Subchronic Systemic Administrations of Escitalopram and Vortioxetine on the Functions of 5-HT1AR and 5-HT3R in the mPFC and on the Extracellular GABA Level in the mPFC.

The subchronic systemic administration of escitalopram (5 mg/kg) did not affect the extracellular GABA level in the mPFC, whereas the subchronic systemic administration of vortioxetine (2.5 mg/kg) weakly increased the extracellular GABA level in the mPFC (Figure 4A,C). Perfusion with 10 µM SR57227 into the mPFC increased the regional extracellular GABA level (Figure 4A,C), whereas perfusion with 50 µM BP554 (5-HT1AR agonist) into the mPFC decreased it (Figure 4B,D).

The stimulatory effects of perfusion with 100 µM SR57227 into the mPFC on regional GABA release was reduced by subchronic escitalopram administration (F_SR_ (2, 15) = 11.7 (*p* < 0.05), F_Time_ (5.5, 83.1) = 113.7 (*p* < 0.05), F_SR*Time_ (11.1, 83.1) = 38.1 (*p* < 0.05)) (Figure 4A,C), but not by subchronic vortioxetine administration (F_SR_ (2, 15) = 20.3 (*p* < 0.05), F_Time_ (4.4, 66.6) = 118.4 (*p* < 0.05), F_SR*Time_ (8.9,66.6) = 28.4 (*p* < 0.05)) (Figure 4A,C). Contrary to SR57227, the inhibitory effects of perfusion with 50 µM BP554 into the mPFC on regional GABA release were inhibited by the subchronic administrations of both escitalopram (F_BP_ (2, 15) = 8.6 (*p* < 0.05), F_Time_ (9, 135) = 51.1 (*p* < 0.05), F_BP*Time_ (18, 135) = 27.0 (*p* < 0.05)) and vortioxetine (F_BP_ (2, 15) = 10.0 (*p* < 0.05), F_Time_ (9, 135) = 61.8 (*p* < 0.05), F_BP*Time_ (18, 135) = 27.8 (*p* < 0.05)) (Figure 4B,D).

### 2.3. Effects of Subchronic Systemic Administrations of Escitalopram and Vortioxetine on the Function of 5-HT1AR and 5-HT3R in the DRN and on Extracellular Levels of 5-HT and GABA in the mPFC (Study-3)

**Figure 5:**
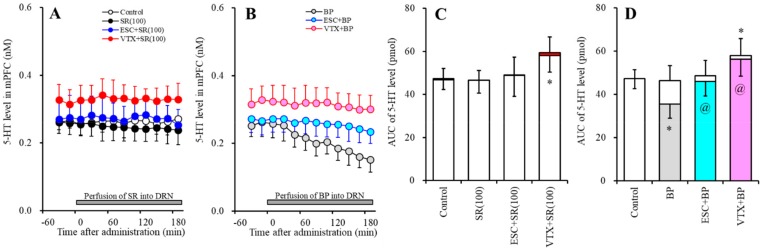
Effects of subchronic systemic administrations of escitalopram and vortioxetine on 5-HT release in the mPFC mediated by functions of 5-HT3R and 5-HT1AR in the DRN. (**A**,**B**) indicate the effects of perfusion with 100 µM SR57227 (SR: 5-HT3R agonist) and 50 µM BP554 (BP: 5-HT1AR agonist) into the DRN on the extracellular 5-HT level in the mPFC, after the subchronic systemic administrations of escitalopram and vortioxetine, respectively. Ordinates: mean ± SD (*n* = 6) of the extracellular 5-HT level (nM); abscissa: time after administration of SR57227 or BP554 (min). Gray bars indicate perfusion with SR57227 or BP554 into the DRN. Microdialysis was conducted to measure the releases of 5-HT and GABA. (**C**,**D**) indicate the AUC value of the extracellular 5-HT level (pmol) after perfusion with SR57227 or BP554 from 20 to 180 min, based on (**A**,**B**), respectively. Opened columns represent the AUC values before perfusion with SR57227 or BP554. * *p* < 0.05, relative to control (in (**A**)), and ^@^ *p* < 0.05, relative to SR57227 (in (**A**)) or BP554 (in (**B**)) by MANOVA with Tukey’s post hoc test.

**Figure 6:**
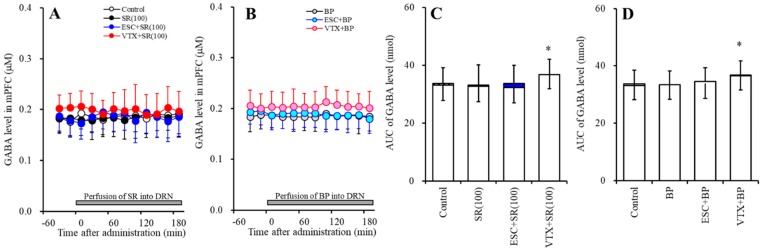
Effects of subchronic systemic administrations of escitalopram and vortioxetine on GABA release in the mPFC mediated by functions of 5-HT3R and 5-HT1AR in the DRN. (**A**,**B**) indicate the effects of perfusion with 100 µM SR57227 (SR: 5-HT3R agonist) and 50 µM BP554 (BP: 5-HT1AR agonist) into the DRN on the extracellular GABA level in the mPFC, after the subchronic systemic administrations of escitalopram and vortioxetine, respectively. Ordinates: mean ± SD (*n* = 6) of the extracellular GABA level (µM); abscissa: time after administration of SR57227 or BP554 (min). Gray bars indicate perfusion with SR57227 or BP554 into the DRN. Microdialysis was conducted to measure the releases of 5-HT and GABA. (**C**,**D**) indicate the AUC value of the extracellular GABA level (nmol) after perfusion with SR57227 or BP554 from 20 to 180 min, based on (**A**,**B**), respectively. Opened columns represent the AUC values before perfusion with SR57227 or BP554. * *p* < 0.05, relative to control (in (**A**)) by MANOVA with Tukey’s post hoc test.

Study-3 was designed to determine the effects of subchronic systemic administrations of effective doses of escitalopram (5 mg/kg/d) and vortioxetine (2.5 mg/kg/d) for 7 d on serotonergic and GABAergic transmissions in the mPFC associated with the 5-HT1AR and 5-HT3R in the DRN. After a 24 h stop of subchronic administrations of escitalopram and vortioxetine, a perfusion medium in the mPFC and the DRN was commenced with MRS. After confirming the stabilization of extracellular levels of 5-HT and GABA in the mPFC, the perfusate in the DRN was switched to MRS containing 100 µM SR57227 or 50 µM BP554 (Figure 5 and Figure 6).

#### 2.3.1. Effects of Subchronic Systemic Administrations of Escitalopram and Vortioxetine on Functions of 5-HT1AR and 5-HT3R in the DRN on the Extracellular 5-HT Level in the mPFC

Perfusion with 50 µM BP554 into the DRN reduced the extracellular 5-HT level in the mPFC (Figure 5B,D), whereas the inhibitory effects of BP554 were inhibited by subchronic administrations of both escitalopram (F_BP_ (2, 15) = 5.8 (*p* < 0.05), F_Time_ (9, 135) = 22.2 (*p* < 0.05), F_BP*Time_ (18, 135) = 11.9 (*p* < 0.05)) and vortioxetine (F_BP_ (2, 15) = 14.4 (*p* < 0.05), F_Time_ (6.5, 97.0) = 18.2 (*p* < 0.05), F_BP*Time_ (12.9, 97.0) = 12.0 (*p* < 0.05)] (Figure 5B,D). Perfusion with 100 µM SR57227 into the DRN did not affect the extracellular 5-HT level in the mPFC (Figure 5A,C). After the subchronic administrations of escitalopram and vortioxetine, perfusion with 100 µM SR57227 into the DRN also did not affect the extracellular 5-HT level in the mPFC (Figure 5A,C).

#### 2.3.2. Effects of Subchronic Systemic Administrations of Escitalopram and Vortioxetine on the Functions of 5-HT1AR and 5-HT3R in the DRN and on the Extracellular GABA Level in the mPFC

The subchronic systemic administration of escitalopram (5 mg/kg) did not affect the extracellular GABA level in the mPFC, whereas the subchronic systemic administration of vortioxetine (2.5 mg/kg) weakly increased the extracellular GABA level in the mPFC (Figure 6A,C). Neither perfusion with 100 µM SR57227 nor that with 50 µM BP554 into the DRN affected the extracellular GABA level in the mPFC (Figure 6). After the subchronic administrations of escitalopram and vortioxetine, neither perfusion with 100 µM SR57227 nor that with 50 µM BP554 into the DRN affected the extracellular GABA level in the mPFC (Figure 6).

### 2.4. Effects of Subchronic Systemic Administrations of Escitalopram and Vortioxetine on Releases of 5-HT and GABA in the mPFC Induced by Local MK801 Administration into the DRN and RTN (Study-4)

**Figure 7:**
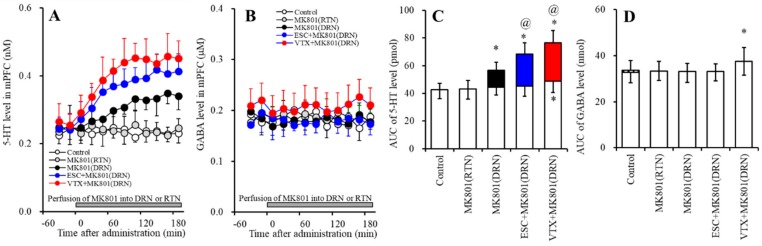
Effects of subchronic systemic administrations of escitalopram and vortioxetine on releases of 5-HT and GABA in the mPFC induced by local MK801 administration into the DRN and RTN. (**A**,**B**) indicate the effects of perfusion with 5 µM MK801 (NMDA-R antagonist) into the RTN and DRN on extracellular levels of 5-HT and GABA in the mPFC, after the subchronic systemic administrations of escitalopram and vortioxetine, respectively. Ordinates: mean ± SD (*n* = 6) of extracellular levels of 5-HT (nM) and GABA level (µM); abscissa: time after administration of MK801 (min). Gray bars indicate perfusion with MK801 into the RTN or DRN. Microdialysis was conducted to measure the releases of 5-HT and GABA. (**C**,**D**) indicate the AUC value of the extracellular levels of 5-HT (pmol) and GABA level (nmol) after perfusion with MK801 from 20 to 180 min, based on (**A**,**B**), respectively. Opened columns represent the AUC values before perfusion with MK801. * *p* < 0.05, relative to control, and ^@^ *p* < 0.05, relative to MK801 (DRN) by MANOVA with Tukey’s post hoc test.

Study-4 was designed to determine the effects of subchronic systemic administrations of effective doses of escitalopram (5 mg/kg/d) and vortioxetine (2.5 mg/kg/d) for 7 d on serotonergic and GABAergic transmissions in the mPFC induced by the inhibition of NMDA-R in the DRN using 5 µM MK801 [25,26,27]. After a 24 h stop of subchronic administrations of escitalopram and vortioxetine, a perfusion medium in the mPFC, the DRN, and the RTN was commenced with MRS. After confirming the stabilization of levels of 5-HT and GABA in the mPFC, the perfusion medium in the DRN or RTN was switched to MRS containing 5 µM MK801 (Figure 7).

#### 2.4.1. Effects of Subchronic Systemic Administrations of Escitalopram and Vortioxetine on 5-HT Release in the mPFC Induced by Local MK801 Administration into the DRN

Perfusion with 5 µM MK801 into the DRN increased the extracellular 5-HT level in the mPFC (F_MK_ (1,10) = 16.0 (*p* < 0.05), F_Time_ (6.6,65.7) = 11.6 (*p* < 0.05), F_MK*Time_ (6.6,65.7) = 15.2 (*p* < 0.05)); however, perfusion with 5 μM MK801 into the RTN did not affect the extracellular 5-HT level in the mPFC (Figure 7A,C). The DRN MK801-induced 5-HT release in the mPFC was enhanced by subchronic administrations of escitalopram (F_MK_ (2, 15) = 19.7 (*p* < 0.05), F_Time_ (9, 135) = 53.9 (*p* < 0.05), F_MK*Time_ (18, 135) = 18.4 (*p* < 0.05)) and vortioxetine (F_MK_ (2, 15) = 32.2 (*p* < 0.05), F_Time_ (4.8, 72.0) = 47.9 (*p* < 0.05), F_MK*Time_ (9.6, 72.0) = 17.8 (*p* < 0.05)) (Figure 7A,C).

#### 2.4.2. Effects of Subchronic Systemic Administrations of Escitalopram and Vortioxetine on GABA Release in the mPFC Induced by Local MK801 Administration into the DRN

Perfusion with 5 µM MK801 into both the DRN and the RTN did not affect the extracellular GABA level in the mPFC (Figure 7B,D). After the subchronic administrations of escitalopram and vortioxetine, perfusion with 5 µM MK801 into the DRN also did not affect the extracellular GABA level in the mPFC (Figure 7B,D).

## 3. Discussion

**Figure 8:**
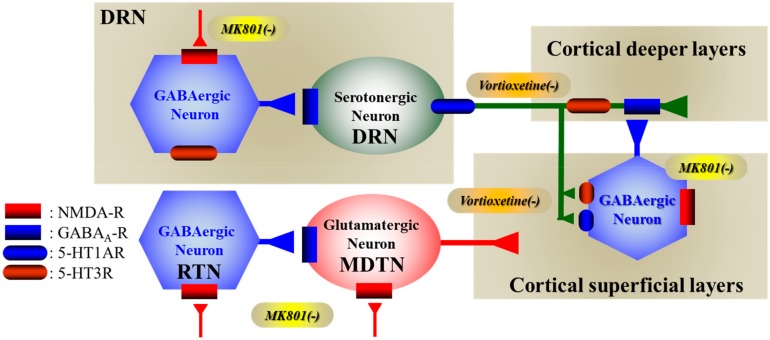
Proposed hypothesis for the extended neural circuitry involved in mesocortical (DRN–mPFC) serotonergic connectivity and their regulation systems. Serotonergic neurons in the DRN receive GABAergic inhibition from GABAergic interneurons in the DRN [28,34]. In the DRN, the GABAergic interneuron in the DRN is predominantly regulated by excitatory NMDA-R and 5-HT3R, whereas the serotonergic neuron in the DRN is regulated by inhibitory GABA_A_-R and 5-HT1AR [28,34]. The released 5-HT in the mPFC activates 5-HT3R on the serotonergic terminals and both 5-HT1AR and 5-HT3R on the GABAergic neurons in the mPFC. Contrary to DRN, neither RTN nor MDTN receive serotonergic regulation associated with 5-HT1AR or 5-HT3R. Furthermore, the thalamocortical glutamatergic terminals (from RTN-MDTN to mPFC) do not contact with serotonergic terminals in the mPFC.

The present study demonstrated the presence of several regulatory systems in the mesocortical (DRN–mPFC) serotonergic pathways that control releases of GABA and serotonin in the mPFC and DRN. According to the results in this study and published neural circuits, our proposed hypothesis regards the mechanisms of clinical action of vortioxetine associated with 5-HT3R and NMDA-R in Figure 8.

### 3.1. Regulation Mechanisms Asociated with 5-HT1AR in the Mesocortical Serotonergic Pathway

Desensitization/downregulation of 5-HT1AR has been considered to be implicated in the clinical delay of monoaminergic antidepressants [35]. Indeed, in the present study, subchronic administrations of both escitalopram and vortioxetine for seven days desensitized 5-HT1AR in both the mPFC and the DRN, since the inhibitory effect of perfusion with BP554 (5-HT1AR agonist) into the mPFC and DRN on regional releases of 5-HT was inhibited by subchronic administrations of these antidepressants. 5-HT1AR exists as two populations: an autoreceptor, which is expressed in the somatodendritic/presynaptic regions of the serotonergic neuron in the DRN, and an heteroreceptor, which is widely (including mPFC) expressed in the postsynaptic region of various neurons [35]. Electrophysiological studies have demonstrated that the inhibitory 5-HT1AR response in serotonergic neurons was more than 90% in the DRN, whereas the inhibitory 5-HT1AR response in GABAergic neurons was about 15% in the DRN [36]. Taken together with these findings, our data suggest that 5-HT1AR in the DRN predominantly inhibits serotonergic neurons rather than GABAergic neurons. Contrary to the DRN, local administration of BP554 into the mPFC decreased regional releases of both GABA and 5-HT. The reduced GABA release in the mPFC induced by local BP554 administration into the mPFC is generated by the activation of postsynaptic 5-HT1AR (heteroreceptor) in the mPFC [15,33,37]. The releases of other monoaminergic transmitters, dopamine and norepinephrine, are increased by the activation of 5-HT1AR via partially GABAergic disinhibition [38,39]. Additionally, the inhibition of NMDA-R in the mPFC also increases regional monoamine release via GABAergic disinhibition [37,38,39,40]. Therefore, the reduced 5-HT release in the mPFC induced by local BP554 administration into the mPFC is generated by direct presynaptic 5-HT1AR activation. This hypothesis is supported by previous studies, which show that the local administration of a 5-HT1AR agonist, 8-OH-DPAT, into the mPFC decreased regional 5-HT release, and the reduced 5-HT release in the mPFC induced by systemic 8-OH-DPAT administration was attenuated by the local administration of a 5-HT1AR antagonist, WAY100635, into the mPFC [41,42]. Therefore, the present study demonstrated that subchronic administrations of both SET inhibitors, escitalopram and vortioxetine, generated the desensitization of somatodendritic 5-HT1AR (autoreceptor) in the DRN, postsynaptic 5-HT1AR (heteroreceptor), and presynaptic 5-HT1AR in the mPFC.

### 3.2. Regulation Mechanisms Associated with 5-HT3R in the Mesocortical Serotonergic Pathway

5-HT3R expresses in the cortex, but not in the DRN [18]. Interestingly, in the mPFC, 5-HT3R predominantly expresses in GABAergic neurons in superficial layers of the mPFC [13]. These types of GABAergic neurons project to monoaminergic terminals in the deeper layers of the mPFC [37,38,40]. Based on these expression patterns, 5-HT3R in the mPFC suppresses neuronal activities in the mPFC via activation of GABAergic inhibition, whereas, conversely, inhibition of 5-HT3R enhances neuronal activities in the mPFC via GABAergic disinhibition [13]. In the present study, local administration of ondansetron (5-HT3R antagonist) into the mPFC increased and decreased regional basal releases of 5-HT and GABA, respectively. Furthermore, local ondansetron administration into the mPFC increased escitalopram-induced 5-HT elevation via GABAergic disinhibition in the mPFC. Contrary to ondansetron, local administration of 10 µM SR57227 (5-HT3R agonist) into the mPFC increased regional GABA release without affecting basal 5-HT release, whereas perfusion with 100 µM SR57227 increased releases of both 5-HT and GABA. The present study cannot explain the mechanisms of these paradoxes regarding responses between lower (10 µM) and higher (100 µM) concentrations of SR527227; however, generally, GABAergic interneurons are more sensitive, since the resting membrane potential of GABAergic interneurons is more positive (from −50 to −60 mV) than that of serotonergic and glutamatergic neurons [36,43]. Taken together with previous findings, the present demonstrations suggest that 5-HT3R might activate releases of 5-HT and GABA via presynaptic serotonergic terminal and postsynaptic GABAergic neurons in the mPFC, respectively.

Long-term exposure to agonists also generates 5-HT3R desensitization/downregulation [44]; however, the effects of subchronic administrations of antidepressants on the function of 5-HT3R remain to be clarified. In accordance with our expectations, in the present study, the subchronic administration of escitalopram generated 5-HT3R desensitization, but that of vortioxetine did not. These discrepancies between the subchronic administrations of escitalopram and vortioxetine suggest that elevation of the extracellular 5-HT level generates desensitization of 5-HT3R, but the 5-HT3R antagonism of vortioxetine probably prevents 5-HT3R desensitization induced by the elevation of the extracellular 5-HT level via SET inhibition. A recent behavioral study demonstrated that 5-HT3R knockout mice exhibited an antidepressant-like phenotype [19]. In spite of lower CNS penetration, ondansetron improves depressive-like behaviours of several depressive models [20,45]. Moreover, inhibition or desensitization of 5-HT3R enhances low-dose action of citalopram [19]. Taken together with these previous behavioral and electrophysiological findings, vortioxetine probably contributes to rapid antidepressant synaptic dynamisms via its 5-HT3R antagonism (direct inhibition of 5-HT3R).

### 3.3. Effects of Vortioxetine on Activated Transmission Induced by NMDA-R Attenuation

Recent psychopharmacology studies have explored the NMDA-R function in physiological and pathological conditions. An NMDA-R antagonist, esketamine, was approved by the FDA in 2019 as a rapid actor against antidepressant-resistant major depressive disorder [21,22]; however, NMDA-R antagonists generate and exacerbate schizophrenia-like cognitive dysfunction in healthy individuals and patients with schizophrenia, respectively [23,24]. Notably, various pharmacodynamic studies have explored the mechanisms of the psychotropic and rapid-acting antidepressant-like actions of ketamine [46,47]. The inhibition of NMDA-R activates glutamatergic transmission via GABAergic disinhibition in the cortex and subcortical regions, including the RTN and DRN [25,28,34,37,38,39,40,48,49,50]. Indeed, in this study, MK801 (NMDA-R antagonist) drastically increased 5-HT release via GABAergic disinhibition in the DRN [28,34].

On the other hand, local administration of NMDA-R antagonist into the mPFC also enhances regional releases of monoamine (dopamine, norepinephrine, and serotonin) through GABAergic disinhibition [37,38,40]. The major frontal monoaminergic terminals are projected to deeper layers in the mPFC, which is regulated by directly inhibitory GABAergic interneurons but indirectly excitatory glutamatergic transmission [37,38,39,40,48]. Contrary to deeper layers, the frontal superficial catecholaminergic terminals are projected from catecholaminergic nuclei; however, serotonergic neurons do not project to superficial layers in the mPFC [38,40]. The thalamocortical glutamatergic pathway (RTN-MDTN to superficial layers of the mPFC), which is one of the responsible pathways regarding NMDA-R attenuation-induced hyperglutamatergic function in the mPFC, activates catecholaminergic terminals in the superficial layers of the mPFC. Therefore, the serotonergic transmission in the superficial layers of the mPFC distinguishes the catecholaminergic transmission [37,38,40], whereas the unique features of the regulation mechanism of serotonergic transmission in the superficial layer of the mPFC remain to be clarified.

In our recent studies, hyperactivation of thalamocortical glutamatergic transmission has been considered as contributing to the cognitive impairment of several neuropsychiatric disorders [25,26,49,50,51]. Under physiological conditions, the MDTN regulates a wide range of cognitive processes, and the enhancement of the sensitivity and reliability of MDTN signaling contributes to the augmentation of flexibility and stability against environmental changes [25,26,27]. Severe GABAergic disinhibition in the MDTN induced by NMDA-R attenuation in the RTN leads to continuous hyperactivated MDTN glutamatergic activities, resulting in the relative deterioration of sensitivity to the input signaling from other regions, which is functionally similar to the disruption of MDTN activity [25,26,27,50,52]. According to the neuronal connectivity in the mPFC, the present study determined the interaction between thalamocortical glutamatergic transmission and mesocortical serotonergic transmission in the mPFC, whereas, unlike catecholamine, hyper thalamocortical glutamatergic transmission induced by inhibition of NMDA-R in the RTN [25,26,49,50] did not affect 5-HT release in the mPFC. Therefore, the regulation mechanism of serotonergic transmission in the mPFC is not identical to that of the transmission of catecholamine (dopamine and norepinephrine). In other words, the pathophysiology of cognitive enhancement associated with serotonergic transmission does not contribute to thalamocortical glutamatergic hyperactivation.

## 4. Materials and Methods

### 4.1. Chemical Agents

Vortioxetine, escitalopram, ondansetron (5-HT3R antagonist), and SR57227 (5-HT3R agonist) were obtained from Cosmo-Bio (Tokyo, Japan). MK801 (noncompetitive NMDA-R antagonist) and BP554 (5-HT1AR agonist) were obtained from Fujifilm-Wako (Osaka, Japan).

All compounds were prepared on the day of the experiment. All drugs were perfused in a modified Ringer’s solution (MRS) made up of (in mM) 145Na^+^, 2.7K^+^, 1.2Ca^2+^, 1.0Mg^2+^, and 154.4Cl^−^, buffered to pH 7.4 with a 2 mM phosphate buffer and a 1.1 mM Tris buffer [53]. Escitalopram, MK801, SR57227, and ondansetron were dissolved in MRS directly. Vortioxetine and BP554 were initially dissolved in dimethyl sulfoxide at 25 mM. The final dimethyl sulfoxide concentration was lower than 0.1% (*v*/*v*).

Previous studies have reported that the minimum effective doses of systemic administrations of vortioxetine and escitalopram on extracellular 5-HT level are 2.5 mg/kg [5] and 5 mg/kg [29], respectively. According to these previous reports, in the present study, to study the acute effects of vortioxetine and escitalopram, each rat was intraperitoneally administered vortioxetine (2.5 mg/kg) or escitalopram (5 mg/kg). To study the subchronic effects of vortioxetine and escitalopram, each rat was subcutaneously administered vortioxetine (2.5 mg/kg/d for 7 d) or escitalopram (5 mg/kg/d for 7 d) using an osmotic pump (2ML_1, Alzet, Cupertino, CA, USA). The present study used concentrations of BP554 (50 µM), ondansetron (10 µM), SR57227 (10 and 100 μM), and MK801 (5 µM) according to previous studies [15,25,26,27,30,31,33,34].

### 4.2. Preparation of the Microdialysis System

All animal care and experimental procedures described in this report complied with the ethical guidelines established by the Institutional Animal Care and Use Committee at Mie University (No. 2019-3 at 24 May 2019). All studies involving animals are reported in accordance with the ARRIVE guidelines for reporting experiments involving animals [54]. A total of 108 rats were used in the experiments described in this manuscript.

Male Sprague-Dawley rats (approximately 250 g, 7−8 weeks old, SLC, Shizuoka, Japan) were maintained in a controlled environment (22 ± 1 °C) on a 12 h dark/light cycle. All rats were weighed before initiation of the study. The rats were anesthetized with 1.8% isoflurane and then placed in a stereotaxic frame for dialysis probe implantation. Concentric direct insertion-type dialysis probes (0.22 mm diameter; Eicom, Kyoto, Japan) were implanted in the mPFC (3 mm exposed membrane: A = +3.2 mm, L = +0.8 mm, V = −5.2 mm, relative to bregma), the reticular thalamic nucleus (RTN: 2 mm exposed membrane: A = −1.4 mm, L = +1.2 mm, V = −7.2 mm, relative to bregma), and the DRN (1 mm exposed membrane: A = −8.2 mm, L = 0.2 mm, V = −6.8 mm, relative to bregma at a lateral angle of 15°) [15,39,55,56]. During recovery and experimentation, the rats were housed individually in cages with food and water ad libitum. Perfusion experiments began 18 h after recovery from isoflurane anesthesia. During the experiments, a rat was placed in an in vivo dialysis system for freely moving animals (Eicom) equipped with a two-channel swivel (TCS2-23; ALS, Tokyo, Japan). The perfusion rate was set at 2 μL/min in all experiments using MRS (composition provided below), and dialysate was collected over 20 min sampling epochs. Extracellular levels of 5-HT and GABA were measured at 8 h after the perfusion started. After baseline recording, the perfusion medium was switched to MRS containing target agents or to the intraperitoneal administration of target agents. Each dialysate sample was injected into the UHPLC apparatus. All samples were obtained from awake, freely moving animals.

After the microdialysis experiments, the brain was removed after cervical dislocation during overdose of isoflurane anesthetization. The locations of the dialysis probes were verified in each animal by histological examination of 200 μm thick brain tissue slices (prepared using a Vibratome 1000; Technical Products International Inc., St. Louis, MO, USA).

### 4.3. Determination of Extracellular Levels of GABA and 5-HT

The GABA level was determined by UHPLC (PU-4185; Jasco, Tokyo, Japan) with fluorescence resonance energy transfer detection (FP-4020; Jasco) after dual derivatization with isobutyryl-l-cysteine and *o*-phthalaldehyde [57]. Each derivative reagent solution was prepared by dissolving isobutyryl-l-cysteine (2 mg) or *o*-phthalaldehyde (1 mg) in 0.1 mL ethanol, followed by the addition of a 0.9 mL sodium borate buffer (0.2 M, pH 9.0). Automated precolumn derivation was conducted by mixing a 5 μL sample, standard, or blank solution with a 5 μL derivative reagent solution in reaction vials for 5 min before injection. The derivatised samples (5 μL) were injected by an autosampler (xLC3059AS; Jasco). The analytical column (YMC Triart C18, particle 1.8 μm, 50 × 2.1 mm; YMC, Kyoto, Japan) was maintained at 45 °C. The flow rate was set at 500 μL/min, and elution was performed using a linear gradient of mobile phases A (0.05 M acetate buffer, pH 5.0) and B (0.05 M acetate buffer containing 30% acetonitrile and 30% methanol, pH 3.5) over 10 min. The excitation/emission wavelengths of the fluorescence detector were set at 280/455 nm [26,34,37,49,51].

The level of 5-HT was determined by UHPLC (xLC3185PU; Jasco) with electrochemical detection (ECD-300; Eicom) by a graphite carbon electrode set to +450 mV (vs. Ag/AgCl reference electrode) [58]. The analytical column (Triart C18, particle 1.8 μm, 30 × 2.1 mm; YMC) was maintained at 40 °C, and the flow rate of the mobile phase was set at 400 μL/min. The mobile phase was made up of a 0.1 acetate buffer containing 1% methanol and 50 mg/L EDTA-2Na (final pH 6.0) [59,60,61]. Where possible, we sought to randomize and blind sample data. In particular, for the determination of extracellular transmitter levels, a sample order was set on the autosampler according to a random number table [25,26,27,34,49,50,51].

### 4.4. Data Analysis

Where possible, we sought to randomize and blind sample data. In particular, for the determination of extracellular transmitter levels, a sample order was set on the autosampler according to a random number table. Drug doses and sample size were selected according to previous studies. According to previous studies, all experiments in this study were designed with equally sized animal groups (*n* = 6), and all values are expressed as mean ± SD. A *p* < 0.05 (two-tailed) was considered statistically significant for all tests. Regional transmitter concentrations were analyzed by Mauchly′s sphericity test followed by multivariate analysis of variance (MANOVA) using BellCurve for Excel ver. 3.20 (Social Survey Research Information Co., Ltd., Tokyo, Japan). When the data did not violate the assumption of sphericity (*p* > 0.05), the F-value of MANOVA was analyzed using sphericity-assumed degrees of freedom; however, if the assumption of sphericity was violated (*p* < 0.05), the F-value was analyzed using Chi-Muller’s corrected degrees of freedom by BellCurve for Excel. When the F-value for the drug factor of MANOVA was significant, the data were finally analyzed by Tukey’s post hoc test using BellCurve for Excel. The transmitter level was expressed as the area under the curve between 20 and 180 min (AUC 20–180 min) after perfusion. All statistical analyses complied with the recommendations on experimental design and analysis in pharmacology [62].

## 5. Conclusions

The present study determined the mechanisms of the multimodal action of vortioxetine on mesocortical and thalamocortical serotonergic transmission associated with 5-HT1AR and 5-HT3R compared with the selective SET inhibitor, escitalopram, in rats. Inhibition of 5-HT3R in the mPFC enhanced regional 5-HT release via GABAergic disinhibition. Acute activation of 5-HT1AR in the DRN and mPFC inhibited 5-HT release in the mPFC via enhancement of inhibitory somatodendritic 5-HT1AR in the DRN and presynaptic 5-HT1AR in the mPFC. Escitalopram subchronically activated mesocortical serotonergic transmission via desensitization of 5-HT1AR in the mPFC and DRN, and of 5-HT3R in the mPFC; however, vortioxetine also subchronically activated mesocortical serotonergic transmission via desensitization of 5-HT1AR in the mPFC and DRN, but not 5-HT3R in the mPFC. These demonstrations, the desensitization of 5-HT1AR with the inhibition of 5-HT3R (without 5-HT3R desensitization), at least partially, contribute to the multimodal antidepressant action of vortioxetine in rats. Furthermore, vortioxetine subchronically enhanced MK801-induced 5-HT release in the mPFC via enhancement of mesocortical serotonergic transmission. Thus, this demonstration suggests that vortioxetine is possibly a candidate antidepressant that can act as an augmentation agent for NMDA-R inhibition therapy.

## Abbreviations

5-HT	serotonin
5-HT1AR	serotonin 5-HT_1A_ receptor
5-HT3R	serotonin 5-HT_3_ receptor
5-HT7R	serotonin 5-HT_7_ receptor
DRN	dorsal raphe nucleus
MDTN	mediodorsal thalamic nucleus
mPFC	medial prefrontal cortex
MRS	modified Ringer’s solution
NMDA	*N*-methyl-d-aspartate
NMDA-R	glutamate/*N*-methyl-d-aspartate (NMDA)/glutamate receptor
OND	ondansetron
RTN	reticular thalamic nucleus
SET	serotonin transporter
UHPLC	ultra-high-performance liquid chromatography

## References

[1] FDA (Federal Drug Administration) Brintillex Prescribing Information (Highlights). http://www.accessdata.fda.gov/drugsatfda_docs/label/2013/204447s000lbl.pdf.

[2] EMA (European Medicines Agency) Brintillex (Vortixetine). https://www.ema.europa.eu/en/documents/smop-initial/chmp-summary-positive-opinion-brintellix_en.pdf.

[3] Guilloux J.P., Mendez-David I., Pehrson A., Guiard B.P., Reperant C., Orvoen S., Gardier A.M., Hen R., Ebert B., Miller S. (2013). Antidepressant and anxiolytic potential of the multimodal antidepressant vortioxetine (Lu AA21004) assessed by behavioural and neurogenesis outcomes in mice. Neuropharmacology.

[4] Zohar J., Nutt D.J., Kupfer D.J., Moller H.J., Yamawaki S., Spedding M., Stahl S.M. (2014). A proposal for an updated neuropsychopharmacological nomenclature. Eur. Neuropsychopharmacol. J. Eur. Coll. Neuropsychopharmacol..

[5] Mork A., Pehrson A., Brennum L.T., Nielsen S.M., Zhong H., Lassen A.B., Miller S., Westrich L., Boyle N.J., Sanchez C. (2012). Pharmacological effects of Lu AA21004: A novel multimodal compound for the treatment of major depressive disorder. J. Pharmacol. Exp. Ther..

[6] Jensen J.B., du Jardin K.G., Song D., Budac D., Smagin G., Sanchez C., Pehrson A.L. (2014). Vortioxetine, but not escitalopram or duloxetine, reverses memory impairment induced by central 5-HT depletion in rats: Evidence for direct 5-HT receptor modulation. Eur. Neuropsychopharmacol. J. Eur. Coll. Neuropsychopharmacol..

[7] Du Jardin K.G., Jensen J.B., Sanchez C., Pehrson A.L. (2014). Vortioxetine dose-dependently reverses 5-HT depletion-induced deficits in spatial working and object recognition memory: A potential role for 5-HT1A receptor agonism and 5-HT3 receptor antagonism. Eur. Neuropsychopharmacol. J. Eur. Coll. Neuropsychopharmacol..

[8] Pehrson A.L., Cremers T., Betry C., van der Hart M.G., Jorgensen L., Madsen M., Haddjeri N., Ebert B., Sanchez C. (2013). Lu AA21004, a novel multimodal antidepressant, produces regionally selective increases of multiple neurotransmitters—A rat microdialysis and electrophysiology study. Eur. Neuropsychopharmacol. J. Eur. Coll. Neuropsychopharmacol..

[9] Kelliny M., Croarkin P.E., Moore K.M., Bobo W.V. (2015). Profile of vortioxetine in the treatment of major depressive disorder: An overview of the primary and secondary literature. Clin. Risk Manag..

[10] Svob Strac D., Pivac N., Muck-Seler D. (2016). The serotonergic system and cognitive function. Transl. Neurosci..

[11] Bang-Andersen B., Ruhland T., Jorgensen M., Smith G., Frederiksen K., Jensen K.G., Zhong H., Nielsen S.M., Hogg S., Mork A. (2011). Discovery of 1-[2-(2,4-dimethylphenylsulfanyl)phenyl]piperazine (Lu AA21004): A novel multimodal compound for the treatment of major depressive disorder. J. Med. Chem..

[12] Mork A., Montezinho L.P., Miller S., Trippodi-Murphy C., Plath N., Li Y., Gulinello M., Sanchez C. (2013). Vortioxetine (Lu AA21004), a novel multimodal antidepressant, enhances memory in rats. Pharmacol. Biochem. Behav..

[13] Leiser S.C., Li Y., Pehrson A.L., Dale E., Smagin G., Sanchez C. (2015). Serotonergic Regulation of Prefrontal Cortical Circuitries Involved in Cognitive Processing: A Review of Individual 5-HT Receptor Mechanisms and Concerted Effects of 5-HT Receptors Exemplified by the Multimodal Antidepressant Vortioxetine. ACS Chem. Neurosci..

[14] Yamamura S., Abe M., Nakagawa M., Ochi S., Ueno S., Okada M. (2011). Different actions for acute and chronic administration of mirtazapine on serotonergic transmission associated with raphe nuclei and their innervation cortical regions. Neuropharmacology.

[15] Fukuyama K., Tanahashi S., Hamaguchi T., Nakagawa M., Shiroyama T., Motomura E., Okada M. (2013). Differential mechanisms underlie the regulation of serotonergic transmission in the dorsal and median raphe nuclei by mirtazapine: A dual probe microdialysis study. Psychopharmacology.

[16] Zmudzka E., Salaciak K., Sapa J., Pytka K. (2018). Serotonin receptors in depression and anxiety: Insights from animal studies. Life Sci..

[17] Rajkumar R., Mahesh R. (2010). The auspicious role of the 5-HT3 receptor in depression: A probable neuronal target?. J. Psychopharmacol..

[18] Kilpatrick G.J., Jones B.J., Tyers M.B. (1987). Identification and distribution of 5-HT3 receptors in rat brain using radioligand binding. Nature.

[19] Martin V., Riffaud A., Marday T., Brouillard C., Franc B., Tassin J.P., Sevoz-Couche C., Mongeau R., Lanfumey L. (2017). Response of Htr3a knockout mice to antidepressant treatment and chronic stress. Br. J. Pharm..

[20] Gupta D., Radhakrishnan M., Kurhe Y. (2014). Ondansetron, a 5HT3 receptor antagonist reverses depression and anxiety-like behavior in streptozotocin-induced diabetic mice: Possible implication of serotonergic system. Eur. J. Pharmacol..

[21] U.S. Food, Drug Administration (2019). FDA approves new nasal spray medication for treatment-resistant depression; available only at a certified doctor’s office or clinic. FDA News Release.

[22] Aan Het Rot M., Zarate C.A., Charney D.S., Mathew S.J. (2012). Ketamine for depression: Where do we go from here?. Biol. Psychiatry.

[23] Javitt D.C. (1987). Negative schizophrenic symptomatology and the PCP (phencyclidine) model of schizophrenia. Hillside J. Clin. Psychiatry.

[24] Malhotra A.K., Pinals D.A., Weingartner H., Sirocco K., Missar C.D., Pickar D., Breier A. (1996). NMDA receptor function and human cognition: The effects of ketamine in healthy volunteers. Neuropsychopharmacology.

[25] Fukuyama K., Kato R., Murata M., Shiroyama T., Okada M. (2019). Clozapine Normalizes a Glutamatergic Transmission Abnormality Induced by an Impaired NMDA Receptor in the Thalamocortical Pathway via the Activation of a Group III Metabotropic Glutamate Receptor. Biomolecules.

[26] Okada M., Fukuyama K., Ueda Y. (2019). Lurasidone inhibits NMDA/glutamate antagonist-induced functional abnormality of thalamocortical glutamatergic transmission via 5-HT7 receptor blockade. Br. J. Pharm..

[27] Okada M., Fukuyama K., Kawano Y., Shiroyama T., Suzuki D., Ueda Y. (2019). Effects of acute and sub-chronic administrations of guanfacine on catecholaminergic transmissions in the orbitofrontal cortex. Neuropharmacology.

[28] Okada M., Fukuyama K., Nakano T., Ueda Y. (2019). Pharmacological Discrimination of Effects of MK801 on Thalamocortical, Mesothalamic, and Mesocortical Transmissions. Biomolecules.

[29] Fernandez-Pastor B., Ortega J.E., Grandoso L., Castro E., Ugedo L., Pazos A., Meana J.J. (2017). Chronic citalopram administration desensitizes prefrontal cortex but not somatodendritic alpha2-adrenoceptors in rat brain. Neuropharmacology.

[30] Perez-Palomar B., Mollinedo-Gajate I., Berrocoso E., Meana J.J., Ortega J.E. (2018). Serotonin 5-HT3 receptor antagonism potentiates the antidepressant activity of citalopram. Neuropharmacology.

[31] Dale E., Grunnet M., Pehrson A.L., Frederiksen K., Larsen P.H., Nielsen J., Stensbol T.B., Ebert B., Yin H., Lu D. (2018). The multimodal antidepressant vortioxetine may facilitate pyramidal cell firing by inhibition of 5-HT3 receptor expressing interneurons: An in vitro study in rat hippocampus slices. Brain Res..

[32] Sanchez C., Asin K.E., Artigas F. (2015). Vortioxetine, a novel antidepressant with multimodal activity: Review of preclinical and clinical data. Pharm. Ther..

[33] Tanahashi S., Yamamura S., Nakagawa M., Motomura E., Okada M. (2012). Dopamine D2 and serotonin 5-HT1A receptors mediate the actions of aripiprazole in mesocortical and mesoaccumbens transmission. Neuropharmacology.

[34] Okada M., Fukuyama K., Okubo R., Shiroyama T., Ueda Y. (2019). Lurasidone sub-chronically activates serotonergic transmission via desensitization of 5-HT1A and 5-HT7 receptors in dorsal raphe nucleus. Pharmaceuticals.

[35] Albert P.R., Le Francois B., Millar A.M. (2011). Transcriptional dysregulation of 5-HT1A autoreceptors in mental illness. Mol. Brain.

[36] Gocho Y., Sakai A., Yanagawa Y., Suzuki H., Saitow F. (2013). Electrophysiological and pharmacological properties of GABAergic cells in the dorsal raphe nucleus. J. Physiol. Sci..

[37] Ohoyama K., Yamamura S., Hamaguchi T., Nakagawa M., Motomura E., Shiroyama T., Tanii H., Okada M. (2011). Effect of novel atypical antipsychotic, blonanserin, on extracellular neurotransmitter level in rat prefrontal cortex. Eur. J. Pharmacol..

[38] Yamamura S., Ohoyama K., Hamaguchi T., Kashimoto K., Nakagawa M., Kanehara S., Suzuki D., Matsumoto T., Motomura E., Shiroyama T. (2009). Effects of quetiapine on monoamine, GABA, and glutamate release in rat prefrontal cortex. Psychopharmacology.

[39] Tanahashi S., Ueda Y., Nakajima A., Yamamura S., Nagase H., Okada M. (2012). Novel delta1-receptor agonist KNT-127 increases the release of dopamine and L-glutamate in the striatum, nucleus accumbens and median pre-frontal cortex. Neuropharmacology.

[40] Yamamura S., Ohoyama K., Hamaguchi T., Nakagawa M., Suzuki D., Matsumoto T., Motomura E., Tanii H., Shiroyama T., Okada M. (2009). Effects of zotepine on extracellular levels of monoamine, GABA and glutamate in rat prefrontal cortex. Br. J. Pharm..

[41] Celada P., Puig M.V., Casanovas J.M., Guillazo G., Artigas F. (2001). Control of dorsal raphe serotonergic neurons by the medial prefrontal cortex: Involvement of serotonin-1A, GABA(A), and glutamate receptors. J. Neurosci..

[42] Casanovas J.M., Vilaro M.T., Mengod G., Artigas F. (1999). Differential regulation of somatodendritic serotonin 5-HT1A receptors by 2-week treatments with the selective agonists alnespirone (S-20499) and 8-hydroxy-2-(Di-n-propylamino)tetralin: Microdialysis and autoradiographic studies in rat brain. J. Neurochem..

[43] Hu H., Jonas P. (2014). A supercritical density of Na(+) channels ensures fast signaling in GABAergic interneuron axons. Nat. Neurosci..

[44] Hothersall J.D., Alexander A., Samson A.J., Moffat C., Bollan K.A., Connolly C.N. (2014). 5-Hydroxytryptamine (5-HT) cellular sequestration during chronic exposure delays 5-HT3 receptor resensitization due to its subsequent release. J. Biol. Chem..

[45] Gupta D., Radhakrishnan M., Kurhe Y. (2014). 5HT3 receptor antagonist (ondansetron) reverses depressive behavior evoked by chronic unpredictable stress in mice: Modulation of hypothalamic-pituitary-adrenocortical and brain serotonergic system. Pharmacol. Biochem. Behav..

[46] Duman R.S., Aghajanian G.K., Sanacora G., Krystal J.H. (2016). Synaptic plasticity and depression: New insights from stress and rapid-acting antidepressants. Nat. Med..

[47] Hashimoto K. (2019). Rapid-acting antidepressant ketamine, its metabolites and other candidates: A historical overview and future perspective. Psychiatry Clin. Neurosci..

[48] Tanahashi S., Yamamura S., Nakagawa M., Motomura E., Okada M. (2012). Clozapine, but not haloperidol, enhances glial D-serine and L-glutamate release in rat frontal cortex and primary cultured astrocytes. Br. J. Pharm..

[49] Okada M., Fukuyama K., Kawano Y., Shiroyama T., Ueda Y. (2019). Memantine protects thalamocortical hyper-glutamatergic transmission induced by NMDA receptor antagonism via activation of system xc−. Pharmacol. Res. Perspect..

[50] Fukuyama K., Hasegawa T., Okada M. (2018). Cystine/Glutamate Antiporter and Aripiprazole Compensate NMDA Antagonist-Induced Dysfunction of Thalamocortical L-Glutamatergic Transmission. Int. J. Mol. Sci..

[51] Nakano T., Hasegawa T., Suzuki D., Motomura E., Okada M. (2019). Amantadine Combines Astroglial System Xc(-) Activation with Glutamate/NMDA Receptor Inhibition. Biomolecules.

[52] Vertes R.P., Linley S.B., Hoover W.B. (2015). Limbic circuitry of the midline thalamus. Neurosci. Biobehav. Rev..

[53] Okada M., Wada K., Kiryu K., Kawata Y., Mizuno K., Kondo T., Tasaki H., Kaneko S. (1998). Effects of Ca^2+^ channel antagonists on striatal dopamine and DOPA release, studied by in vivo microdialysis. Br. J. Pharm..

[54] McGrath J.C., Drummond G.B., McLachlan E.M., Kilkenny C., Wainwright C.L. (2010). Guidelines for reporting experiments involving animals: The ARRIVE guidelines. Br. J. Pharm..

[55] Yamamura S., Ohoyama K., Nagase H., Okada M. (2009). Zonisamide enhances delta receptor-associated neurotransmitter release in striato-pallidal pathway. Neuropharmacology.

[56] Tanahashi S., Yamamura S., Nakagawa M., Motomura E., Okada M. (2012). Effect of lamotrigine and carbamazepine on corticotropin-releasing factor-associated serotonergic transmission in rat dorsal raphe nucleus. Psychopharmacology.

[57] Yamamura S., Saito H., Suzuki N., Kashimoto S., Hamaguchi T., Ohoyama K., Suzuki D., Kanehara S., Nakagawa M., Shiroyama T. (2009). Effects of zonisamide on neurotransmitter release associated with inositol triphosphate receptors. Neurosci. Lett..

[58] Okada M., Hirano T., Kawata Y., Murakami T., Wada K., Mizuno K., Kondo T., Kaneko S. (1999). Biphasic effects of zonisamide on serotonergic system in rat hippocampus. Epilepsy Res..

[59] Okada M., Yoshida S., Zhu G., Hirose S., Kaneko S. (2005). Biphasic actions of topiramate on monoamine exocytosis associated with both soluble N-ethylmaleimide-sensitive factor attachment protein receptors and Ca(2+)-induced Ca(2+)-releasing systems. Neuroscience.

[60] Okada M., Hirano T., Mizuno K., Kawata Y., Wada K., Murakami T., Tasaki H., Kaneko S. (1998). Effects of carbamazepine on hippocampal serotonergic system. Epilepsy Res..

[61] Kawata Y., Okada M., Murakami T., Kamata A., Zhu G., Kaneko S. (2001). Pharmacological discrimination between effects of carbamazepine on hippocampal basal, Ca(2+)- and K(+)-evoked serotonin release. Br. J. Pharm..

[62] Curtis M.J., Alexander S., Cirino G., Docherty J.R., George C.H., Giembycz M.A., Hoyer D., Insel P.A., Izzo A.A., Ji Y. (2018). Experimental design and analysis and their reporting II: Updated and simplified guidance for authors and peer reviewers. Br. J. Pharm..

